# Incidental pulmonary embolism in oncologic patients—a systematic review and meta-analysis

**DOI:** 10.1007/s00520-020-05601-y

**Published:** 2020-07-04

**Authors:** Hans-Jonas Meyer, Andreas Wienke, Alexey Surov

**Affiliations:** 1 Department of Diagnostic and Interventional Radiology, University of Leipzig, Leipzig, Germany; 2 Institute of Medical Epidemiology, Biostatistics, and Informatics, Martin-Luther-University Halle-Wittenberg, Halle (Saale), Germany; 3 Department of Diagnostic and Interventional Radiology, University of Magdeburg, Magdeburg, Germany

**Keywords:** Incidental, Pulmonary embolism, Computed tomography, Oncology

## Abstract

**Purpose:**

Incidental pulmonary embolism (IPE) is a common finding on computed tomography (CT). IPE is frequent in oncologic patients undergoing staging CT. The aim of this analysis was to provide the pooled frequency of IPE and frequencies of IPE in different primary tumors.

**Methods:**

MEDLINE, SCOPUS, and EMBASE databases were screened for studies investigating frequency of IPE in oncologic staging CT up to February 2020. Overall, 12 studies met the inclusion criteria and were included into the present study.

**Results:**

The pooled analysis yielded a total of 28,626 patients. IPE was identified in 963 patients (3.36%, 95% CI = 3.15; 3.57). The highest frequency was found in prostate cancer (8.59%, 95%CI = 3.74; 13.44), followed by hepatobiliary carcinoma (6.07%, 95%CI = 3.09; 9.05) and pancreatic cancer (5.65%, 95%CI = 3.54; 7.76). The lowest frequencies were identified in tumors of male reproductive organs (0.79%, 95%CI = 0.21; 1.37) and hematological diseases (1.11% 95%CI = 0.74; 1.48).

**Conclusion:**

The overall frequency of IPE in oncologic patients was 3.36%. There are considerable differences in regard to primary tumors with the highest frequency in prostate cancer and pancreatic and hepatobiliary carcinomas.

## Introduction

Incidental pulmonary embolism (IPE) is defined as an unsuspected filling defect of the pulmonary arteries identified on imaging studies performed for other purposes [1]. Importantly, these embolic events are clinically asymptomatic [1].

It is well-known that oncologic patients are a risk group for thromboembolic events [2]. So, the overall frequency of thromboembolic events is up to 20% in cancer patients [3].

Computer tomographic (CT) pulmonary angiography is the imaging modality of choice to detect or rule out pulmonary embolism with high diagnostic accuracy [4]. Noteworthy, on staging CTs, the detection of pulmonary embolism is lower due to a different contrast phase resulting in poorer contrast of the pulmonary arteries [5]. However, with modern CT scanners, there is no debate that the obtained contrast is sufficient enough in most cases for the diagnosis of pulmonary embolism in clinical routine [5].

An increasing frequency of IPE has been reported in the literature [1]. The main assumed factor for this is the increasing use of CT in clinical routine [1, 6]. The frequency of IPE is mainly studied based upon oncologic patient samples undergoing staging investigations.

The published literature regarding IPE in oncologic patients undergoing staging CT is heterogeneous in regard to investigated primary tumors and utilized CT scanner technology. Presumably, the frequencies of IPE might differ in regard to the primary tumor, as different tumors show different thrombogenic potential and different treatment regimens might have a crucial impact on thromboembolisms in patients. Yet, there is lack of data to identify these differences in oncologic patients.

Thus, the present systematic review and meta-analysis sought to pool studies investigating staging CTs in oncologic patients, which report data about IPE. The aim of this analysis was to provide the pooled frequency of IPE and frequencies in regard to primary tumors.

## Methods

### Literature search and data acquisition

**Fig. 1:**
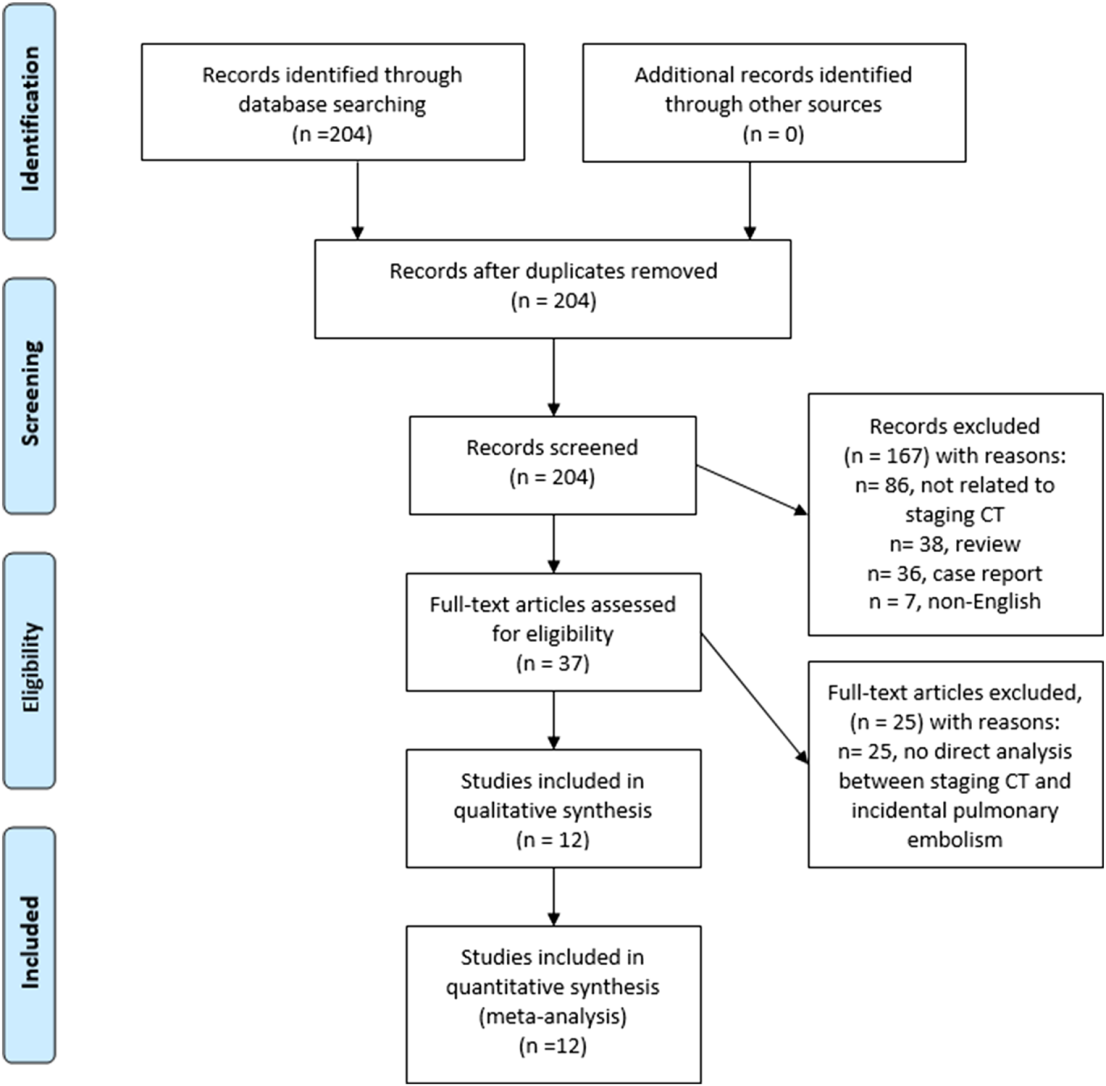
PRISMA flow chart. An overview of the paper acquisition. After exclusion of the identified 204 papers, overall, 12 studies comprising 28,626 patients were suitable for the analysis

MEDLINE database was screened for studies investigating frequency of IPE in oncologic staging CT up to February 2020. The search terms/combinations were as follows: “incidental pulmonary embolism and oncology OR oncologic patient OR staging” (Fig. 1). The Preferred Reporting Items for Systematic Reviews and Meta-Analyses (PRISMA) statement was used for the research [7].

The primary search identified 204 records. The abstracts of the items were checked. Inclusion criteria for this work were as follows: reporting of the frequency of IPE, oncologic staging CT investigating, and written in English. Exclusion criteria were as follows: studies unrelated to the staging CT, studies with incomplete data, not written in English, duplicate publications, review, meta-analysis, and case report articles. After exclusion of not suitable papers, overall 12 studies met the inclusion criteria [8–19].

As the next step, the following data were extracted from the literature: authors, year of publication, study design, number of patients/tumors, tumor type, CT scanner type, and frequency of IPE.

The primary endpoint of the systematic review was the frequency of IPE. Second endpoint was the frequency of IPE according to primary tumor.

### Meta-analysis

**Fig. 2:**
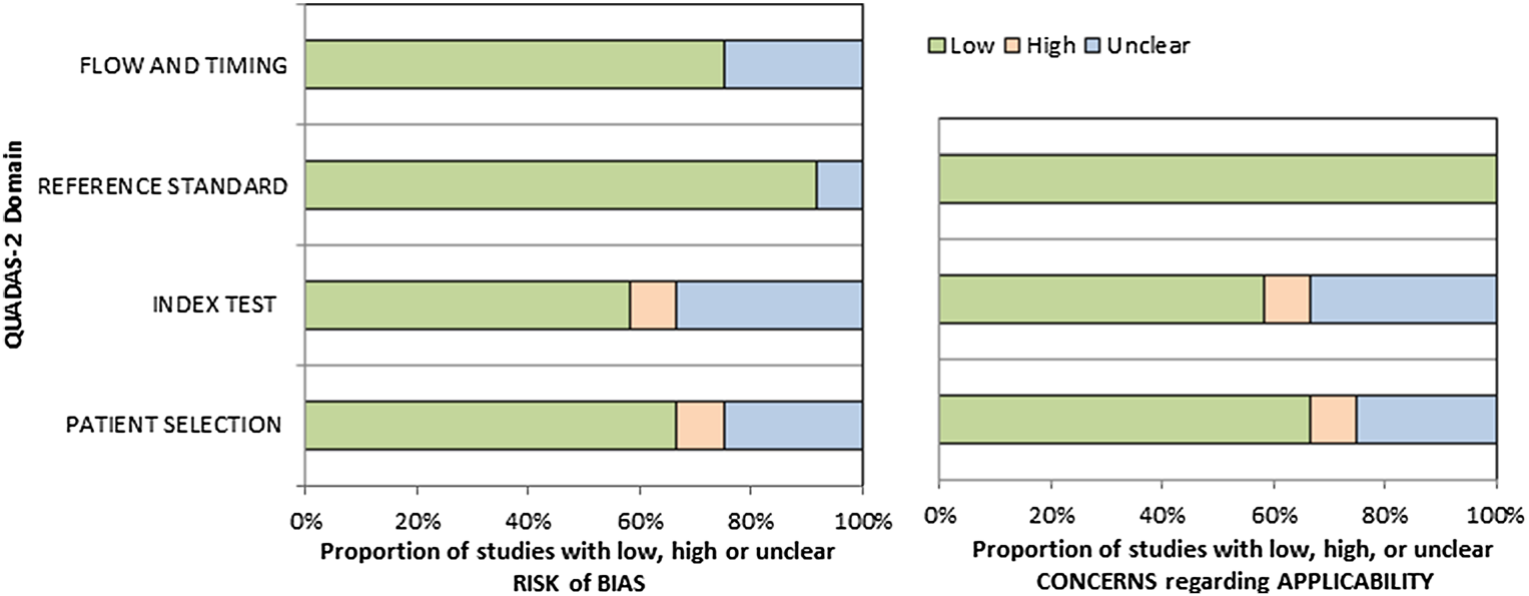
QUADAS-2 quality assessment of the included studies. Most studies showed an overall low risk of bias

The methodological quality of the identified 12 studies was checked according to the Quality Assessment of Diagnostic Accuracy Studies (QUADAS-2) instrument [20] independently by two observers (A.S. and H.J.M.) (Fig. 2). Every paper was tested for patient selection, index test, reference standard, and flow and timing. The resulting overall risk of bias was low. Only one study showed a high risk of bias for index test evaluation and patient selection. An unclear risk of bias was identified for 3 studies for patient selection, 4 studies for index test, 1 study for reference standard, and 3 studies for flow and timing.

The meta-analysis was undertaken by using RevMan (RevMan 2014, the Cochrane Collaboration Review Manager Version 5.3). Because of the heterogeneous conditions in the different studies, a random-effects meta-analysis was performed, which assumed that the study effects vary randomly from study to study. The extent of variation among these study effects observed in different studies (between-study variance) is referred to as τ^2^ [21]. τ^2^ is the variance of the effect size parameters across the population of studies, and it reflects the variance of the true effect sizes. The standard chi-squared test (Cochran Q test) for statistical heterogeneity tests the statistical hypothesis that the true effects are the same (no heterogeneity) in all the primary studies included in meta-analysis [22]. This statistical test uses a test statistic (Chi^2^) that has a chi-squared distribution on k-1 degrees of freedom (*k* represents the number of studies) under the statistical hypothesis; the corresponding *p* value for the test statistic is given.

The *I*^2^ statistic represents the percentage of the variability in effect estimates that is due to heterogeneity [21]. *I*^2^ is the proportion of observed dispersion of results from different studies included in a meta-analysis that is real, rather than spurious. The *I*^2^ index can be interpreted as the percentage of the total variability in a set of effect sizes due to true heterogeneity (between-studies variability). If *I*^2^ = 0%, this indicates that all variability in effect size estimates is due to sampling error within studies. If *I*^2^ = 50%, it indicates that half of the total variability among effect sizes is caused not by sampling error, but by true heterogeneity between studies. *I*^2^ is a percentage, and its values lie between 0 and 100%. A value of 0% indicates no observed heterogeneity, and larger values show increasing heterogeneity [23, 24]. DerSimonian and Laird random-effects models with inverse-variance weights were used without corrections [25]. The frequency of IPE was calculated with 95% confidence intervals (CI).

## Results

The publication date ranges from 2006 [14] to 2018 [16] (Table 1). Most studies were of retrospective design (10 out of 12, 83.3%). Different CT scanner generations were used in the studies (Table 1).

**Table 1:** Overview of the included studies

Author, year	Study design	Number of patients	Cases with incidental pulmonary embolism, *n* (%)	CT scanners
Aleem et al., 2012	Retrospective	701	9 (1.3)	Unclear
Bach et al., 2014	Retrospective	3270	240 (7.3)	64 slices
Browne et al., 2010	Prospective	407	18 (4.4)	64 slices
Cronin et al., 2007	Retrospective	397	13 (3.3)	Unclear
Deniz et al., 2017	Retrospective	1000	46 (4.6)	16 and 64 slices
Di Nisio et al., 2010	Retrospective	1921	24 (1.3)	Unclear
Douma et al., 2010	Retrospective	838	3 (0.34)	Unclear
Engelke et al., 2006	Retrospective	1869	56 (3.0)	4 and 16 slices
Gladish et al., 2006	Retrospective	403	16 (3.9)	16 and 64 slices
Kilburn et al., 2017	Prospective	3306	117 (3.5)	16 and 64 slices
Myat Moe et al., 2018	Retrospective	731	26 (3.6)	128 slices
Shinagare et al., 2011	Retrospective	13,783	395 (2.87)	4 and 64 slices

Overall, the pooled analysis of 12 studies yielded a total of 28,626 patients. In these patients, 963 IPE cases were identified. The overall frequency of IPE in all patients was 3.36%, 95%CI = 3.15; 3.57.

**Fig. 3:**
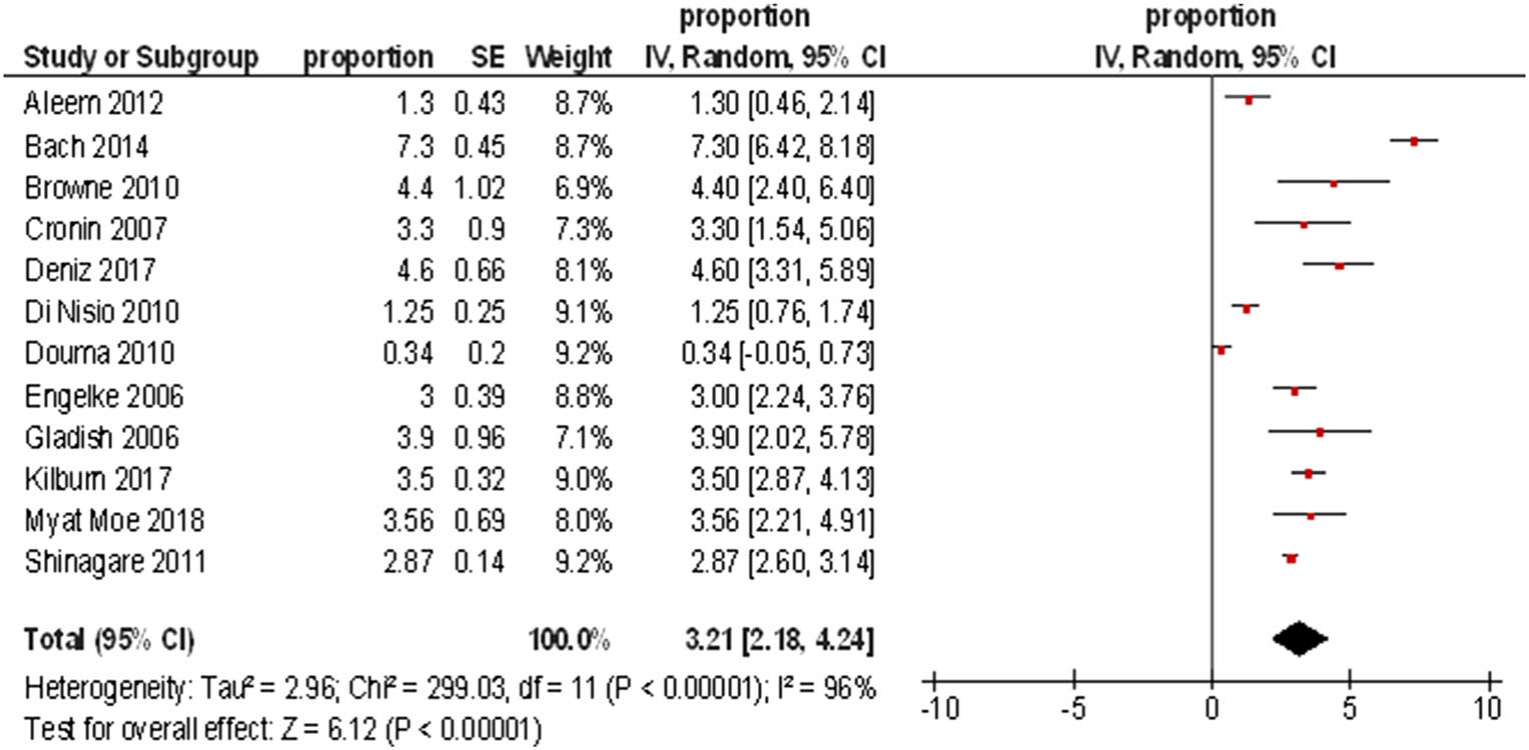
Forrest plots of the frequencies of incidental pulmonary embolism in the total patient sample. The pooled frequency over all studies was 3.2 [95% CI 2.18–4.24]

**Fig. 4:**
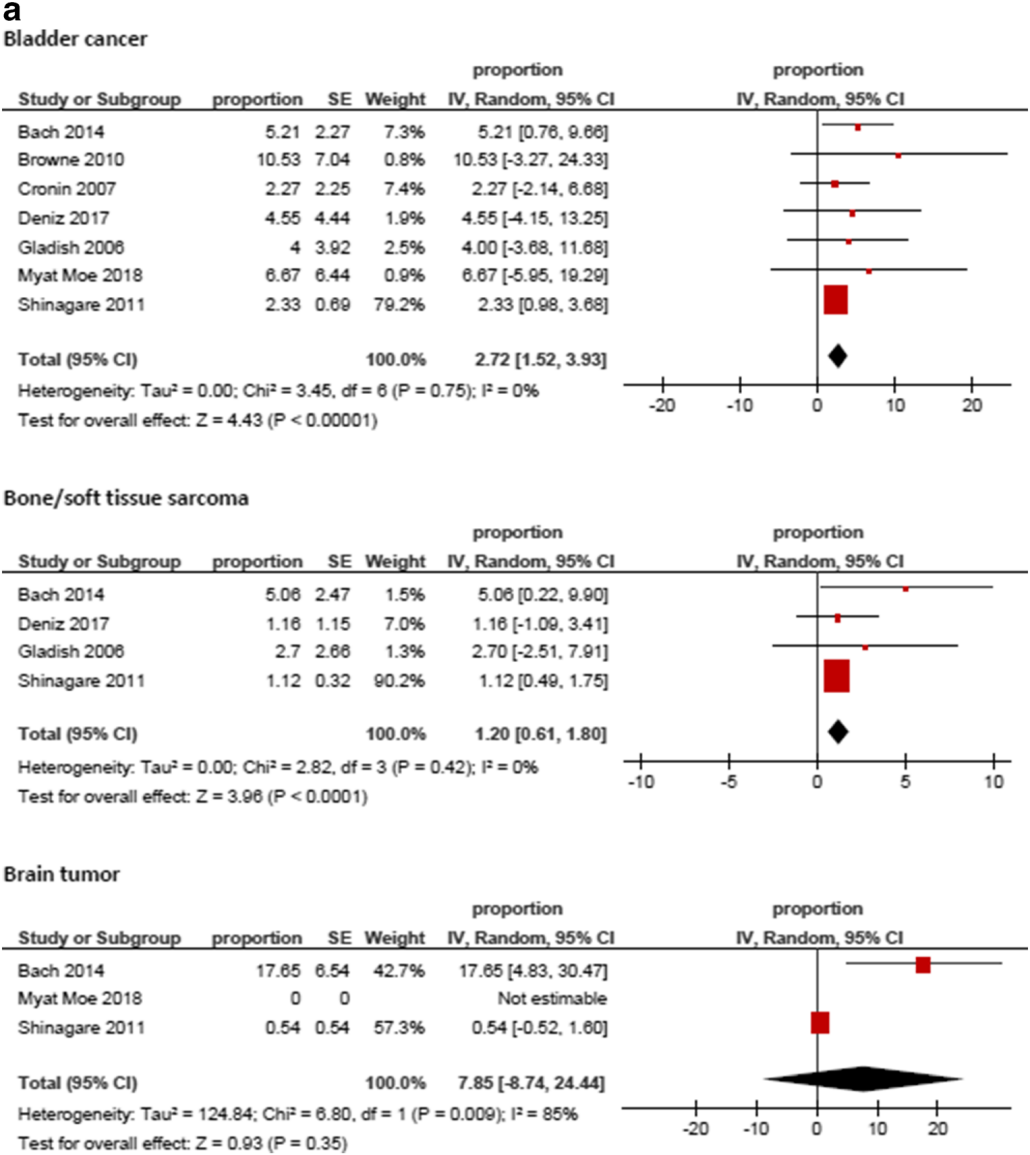

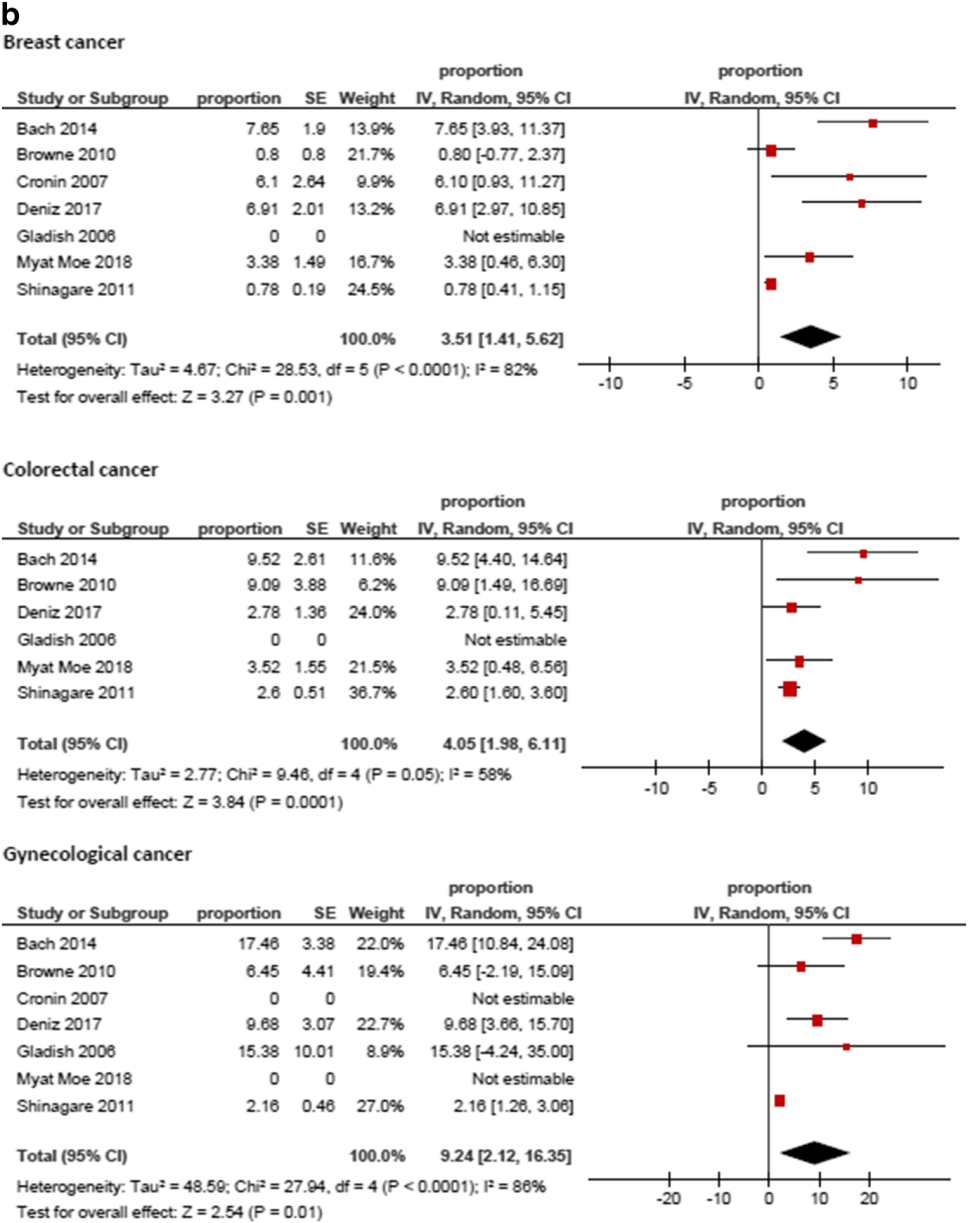

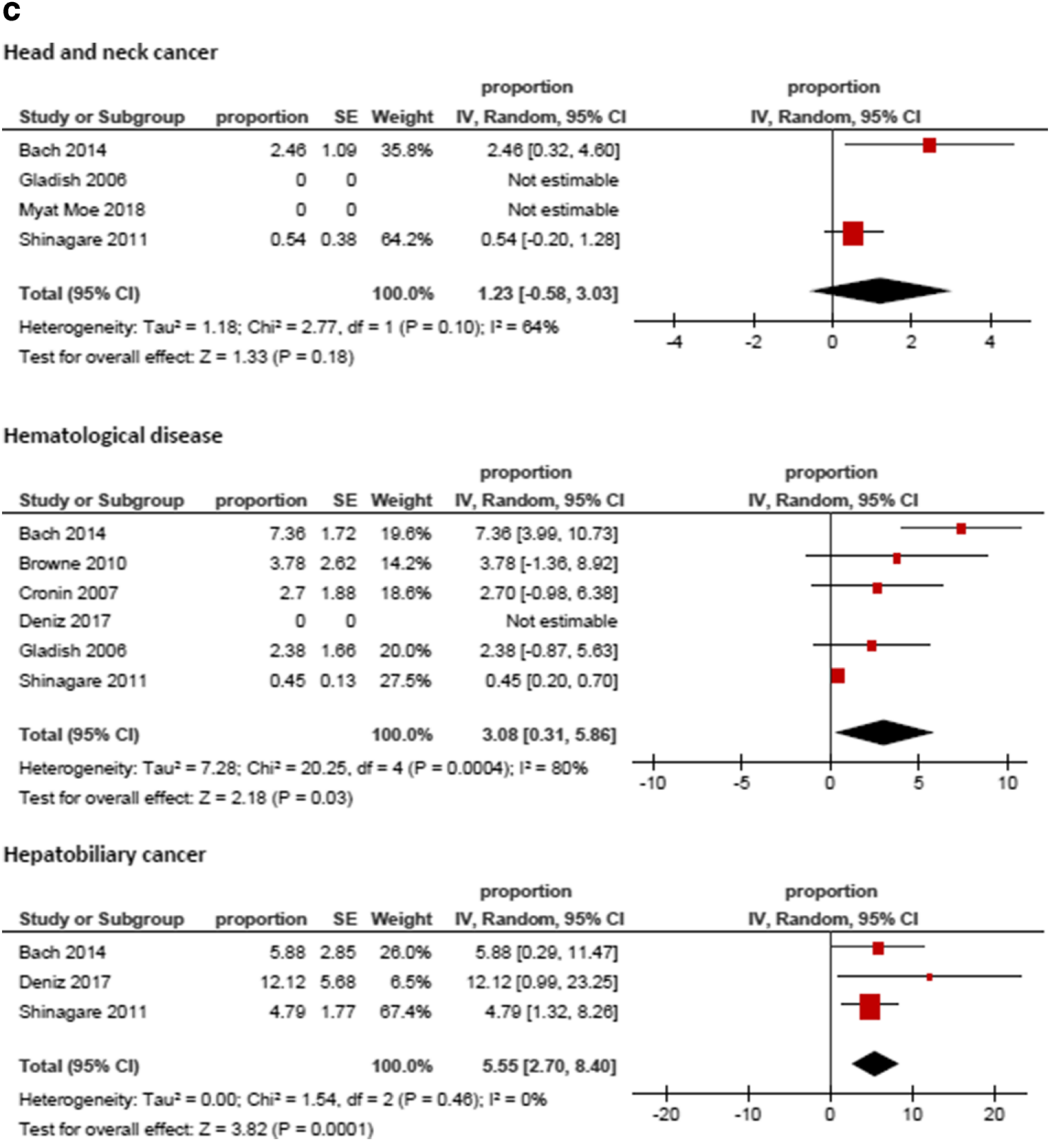

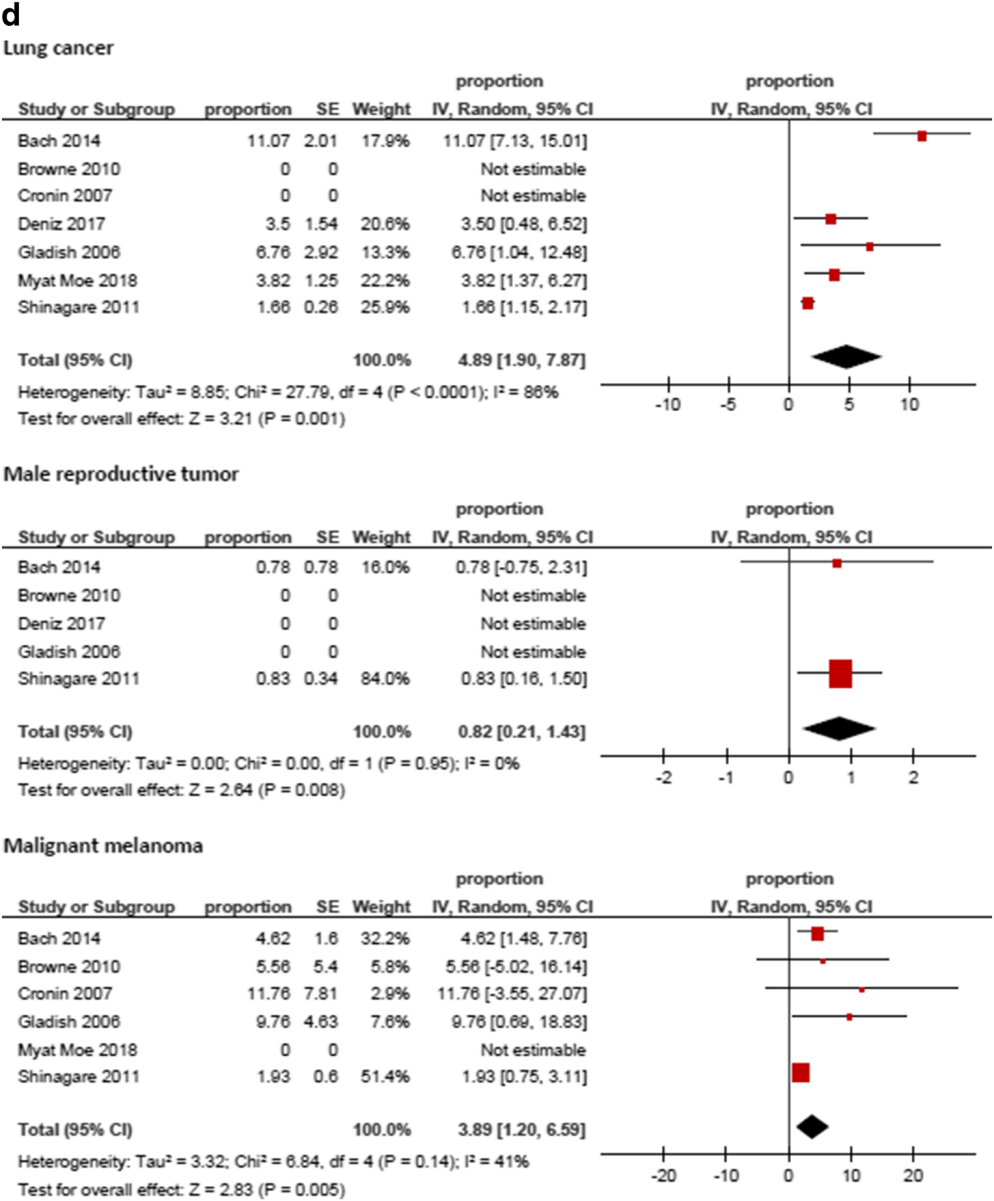

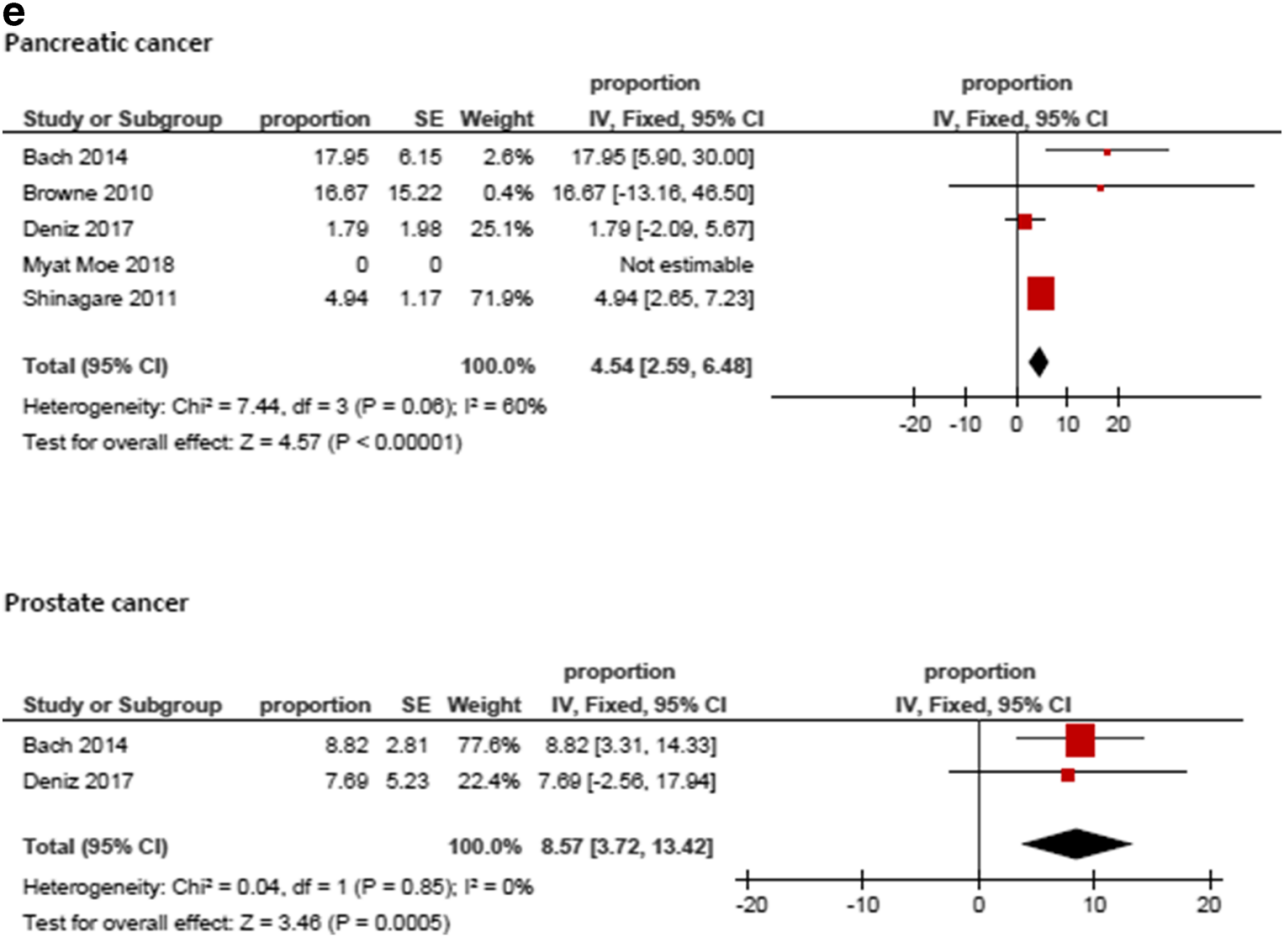
**a**–**e** Forrest plots of the frequencies of incidental pulmonary embolism according to primary tumors. The highest frequency was identified for prostate cancer (8.59%, 95%CI = 3.74; 13.44), followed by hepatobiliary cancer (6.07%, 95%CI = 3.09; 9.05) and pancreatic cancer (5.65%, 95%CI = 3.54; 7.76). The lowest frequencies were identified for tumors of male reproductive organs (0.79%, 95%CI = 0.21; 1.37) and malignant hematological diseases (1.11%, 95%CI = 0.74; 1.48). For studies without any event of incidental pulmonary embolism the frequency could not be included into the meta-analysis

Figure 3 displays the frequencies of IPE reported by the different studies. The highest frequency was identified for prostate cancer (8.59%, 95%CI = 3.74; 13.44), followed by hepatobiliary cancer (6.07%, 95%CI = 3.09; 9.05) and pancreatic cancer (5.65%, 95%CI = 3.54; 7.76). The lowest frequencies were identified for tumor of male reproductive organs (0.79%, 95%CI = 0.21; 1.37) and malignant hematological diseases (1.11%, 95%CI = 0.74; 1.48). The frequencies of IPE in regard to the primary tumor are summarized in Fig. 4.

## Discussion

The present meta-analysis calculates the frequency of IPE in oncologic patients undergoing routine staging CT investigation and provides frequencies of IPE in regard to primary tumor.

It is well-known that cancer patients are a risk group for thromboembolic events, which even precedes the cancer diagnosis of 150 days [2]. So, the overall reported frequency of thromboembolic events is up to 20% in cancer patients [3].

PE has a multifactorial etiology with many independent risk factors associated with venous thromboembolism (VTE), such as surgery, trauma, hospitalization, malignant neoplasm with or without chemotherapy, and the use of central venous catheters [26, 27].

Noteworthy, VTE is an independent prognostic factor of mortality in cancer patients. Thus, cancer patients with VTE have a shorter overall survival than cancer patients without VTE at the same tumor stage with the same treatment [26, 28].

The frequency of IPE ranged in previous reports significantly. So far, in a large retrospective study on 3270 patients undergoing staging CTs, the identified frequency of IPE was 7.3% in the overall sample with significant differences in several primary tumors, ranging from 0 to 25% [9]. Primary tumors with the highest frequencies were colonic cancer, lung cancer, renal carcinoma, and tumors of the upper gastrointestinal tract. Moreover, patients with metastasized diseases had a 1.5-fold higher frequency of pulmonary embolism compared to patients with localized tumor stage [9].

In another retrospective study, a lower prevalence of 1.6% was reported in a sample of 731 patients [16]. Interestingly, in this study, all patients had a metastasized stage, which might result in a high frequency of IPE. In a prospective study evaluating the assessment of pulmonary vessels by radiographers, a frequency of 3.5% was identified (117 out of 3306 patients) [19]. These significant differences might mainly be influenced by different primary tumors in the different oncologic centers with diverse focuses of oncologic care. Another reason might be the different scanner technology used in the radiology department with slightly distinctive accuracy in the detection of small embolisms.

In a first systematic review and meta-analysis in 2010 by Dentali et al., a frequency of 2.6% was reported based upon patients undergoing chest CT [29]. The frequency of the present analysis is slightly higher. Possible reasons for this might be better CT technology and different patient selection. The present analysis only included oncologic patients, whereas in the study by Dentali et al., also nononcologic patients were included, which might have consequently a lower frequency of IPE.

There are also controversies of the clinical relevance of IPE. So, IPE is more often located on the segmental and subsegmental level without an occlusion of the vessel [1, 30, 31]. Correspondingly, the total embolic burden in incidental PE is lower than that in symptomatic PE [30–32]. Especially, subsegmental PE is of interest, which was defined as peripheral PE limited to the fifth order pulmonary arteries. So, some authors treat those PE similar as symptomatic PE with anticoagulation, whereas other authors do not begin treatment in those patients [6]. Yet, the characterizing and detection of IPE in clinical routine might be crucial.

CT pulmonary angiography is the clinical diagnostic gold standard with a high diagnostic accuracy with a pooled sensitivity of 90% and a specificity of 88% [32]. It is a well-known fact that staging CTs, acquired with a portal venous or venous phase, have a poorer contrast of the pulmonary vessels, and consequently, the detection rate of small pulmonary embolisms is poorer [5]. However, with modern CT multislice scanners, the detection rate is higher compared to older scanners. Noteworthy, the reliability of diagnosing IPE in oncologic patients is high with an excellent interreader variability in proximal embolisms with a lesser diagnostic accuracy in distal clots [5, 33]. Due to the introduction of dual-energy CT scanner, the detection rate of small pulmonary embolism is further increased [34]. Yet, no study with this modern imaging technique was included in the present analysis.

The highest frequency of IPE was identified in prostate cancer patients. It has been shown previously that patients with prostate cancer are at higher risk of thromboembolic diseases, with the highest risk for those receiving endocrine therapy [35]. Moreover, it was stated that prostate cancer itself, prostate cancer treatments, and selection mechanisms all contribute to an increased risk of thromboembolic events [35]. Beyond that, the high frequency of IPE in the present study might be caused by the fact that prostate cancer staging CTs are mainly performed at the metastasized tumor stage compared to other tumor entities, which harbors in itself a higher risk of IPE.

Higher frequency of IPE was then identified in patients with pancreatic cancer and hepatobiliary cancer. Pancreatic cancer also leads in the frequency of thromboembolism among hospitalized patients compared to other tumor entities with 8.1% [36]. Several biological features of pancreatic cancer were discussed to induce thromboembolic events [37]. So, genes reported to be regulators of coagulopathy comprise activation of oncogenes as KRAS and c-MET and inactivation of tumor suppressor genes such as p53 [37]. Moreover, the complex surgery procedures in curative pancreatic cancer can lead to thromboembolism [38]. In comparison, similar reasons can be discussed for hepatobiliary cancer [39].

The lowest frequency was identified for patients with male reproductive tumors. These patients are most commonly young patients [40]. Thus, these patients have less comorbidity and a resulting lesser risk of thromboembolic events. So, it was reported that 2 of 295 patients with germ cell tumors suffered from arterial thrombosis undergoing chemotherapy [41].

Notably, the included papers in the present analysis were of substantial heterogeneity, which was shown by the high *I*^2^ value of all presented results. In short, important factors can be different tumor entities, different scanner technology, and study design.

There are several limitations of the present analysis to address. Firstly, it is a pooled analysis of retrospective studies with possible known bias. Secondly, the CT scanners used in some of the studies are of older generations like 4 and 16 slice scanners. There are concerns that the frequency of IPE might be lower in these studies due to undetected embolisms compared with better CT technology. So, the true frequency of IPE might be higher than in the presented results. Thirdly, there might be possible publication bias as studies with higher reported IPE frequencies are more likely to be published. Fourthly, only studies in English language were considered suitable for the analysis.

## Conclusions

The overall frequency of IPE in oncologic patients is 3.36%. The highest frequency of IPE is identified in patients with prostate cancer and pancreatic and hepatobiliary carcinomas. This fact should be known for radiologists and oncologists. In patients with malignant diseases, especially with prostate, pancreatic and hepatobiliary cancers, staging CTs should be also evaluated for the presence of IPE because of its clinical importance.
